# Supplementary material for: Modeling glioblastoma heterogeneity as a dynamic network of cell states
Source: Mol Syst Biol. 2021 Sep 16;17(9):e10105. doi: 10.15252/msb.202010105 (PMC8444284; doi:10.15252/msb.202010105)
Supplement: Supplementary file 6 — Source Data for Figure 5 [file MSB-17-e10105-s004.zip › Figure5A_sourcedata/GSEA_3071/hallmarks_stateI.GseaPreranked.1623742531165/HALLMARK_ANGIOGENESIS.html]

Details for gene set HALLMARK\_ANGIOGENESIS[GSEA]

|  || Dataset | state13071 |
| Phenotype | NoPhenotypeAvailable |
| Upregulated in class | na\_pos |
| GeneSet | HALLMARK\_ANGIOGENESIS |
| Enrichment Score (ES) | 0.76614934 |
| Normalized Enrichment Score (NES) | 3.0166056 |
| Nominal p-value | 0.0 |
| FDR q-value | 0.0 |
| FWER p-Value | 0.0 |
Table: GSEA Results Summary

  

Fig 1: Enrichment plot: HALLMARK\_ANGIOGENESIS      
 Profile of the Running ES Score & Positions of GeneSet Members on the Rank Ordered List

  

| PROBE | GENE SYMBOL | GENE\_TITLE | RANK IN GENE LIST | RANK METRIC SCORE | RUNNING ES | CORE ENRICHMENT || 1 | SPP1 |  |  | 0 | 2.808 | 0.1290 | Yes |
| 2 | COL3A1 |  |  | 3 | 2.646 | 0.2500 | Yes |
| 3 | LUM |  |  | 4 | 2.203 | 0.3512 | Yes |
| 4 | S100A4 |  |  | 30 | 1.506 | 0.4132 | Yes |
| 5 | POSTN |  |  | 32 | 1.494 | 0.4816 | Yes |
| 6 | TIMP1 |  |  | 47 | 1.381 | 0.5410 | Yes |
| 7 | COL5A2 |  |  | 48 | 1.373 | 0.6040 | Yes |
| 8 | NRP1 |  |  | 81 | 1.180 | 0.6491 | Yes |
| 9 | ITGAV |  |  | 117 | 1.029 | 0.6863 | Yes |
| 10 | PDGFA |  |  | 119 | 1.015 | 0.7327 | Yes |
| 11 | VCAN |  |  | 197 | 0.833 | 0.7489 | Yes |
| 12 | JAG2 |  |  | 255 | 0.730 | 0.7661 | Yes |
| 13 | FSTL1 |  |  | 505 | 0.504 | 0.7180 | No |
| 14 | JAG1 |  |  | 796 | 0.383 | 0.6526 | No |
| 15 | KCNJ8 |  |  | 1601 | -0.268 | 0.4347 | No |
| 16 | VEGFA |  |  | 1644 | -0.273 | 0.4352 | No |
| 17 | APP |  |  | 2770 | -0.495 | 0.1359 | No |
| 18 | TNFRSF21 |  |  | 3004 | -0.604 | 0.0970 | No |
| 19 | STC1 |  |  | 3349 | -1.043 | 0.0464 | No |
Table: GSEA details [plain text format]

  

Fig 2: HALLMARK\_ANGIOGENESIS: Random ES distribution      
 Gene set null distribution of ES for **HALLMARK\_ANGIOGENESIS**
